# A scale down process for the development of large volume cryopreservation^☆^

**DOI:** 10.1016/j.cryobiol.2014.09.003

**Published:** 2014-12

**Authors:** Peter Kilbride, G. John Morris, Stuart Milne, Barry Fuller, Jeremy Skepper, Clare Selden

**Affiliations:** a Institute of Liver and Digestive Health, Royal Free Hospital Campus, UCL, London NW3 2PF, UK; b Asymptote Ltd., St. John’s Innovation Centre, Cowley Road, Cambridge CB4 0WS, UK; c Department of Surgery, Royal Free Hospital Campus, UCL, London NW3 2PF, UK; d Multi Imaging Centre, Anatomy Building, Downing Site, Cambridge University, CB2 3DY, UK

**Keywords:** ELS, encapsulated liver spheroids, PS, progressive solidification, NS, network solidification, BAL, bioartificial liver device, Large volume cryopreservation, Bioartificial liver, Undercooling, Network solidification, Progressive solidification

## Abstract

The process of ice formation and propagation during cryopreservation impacts on the post-thaw outcome for a sample. Two processes, either network solidification or progressive solidification, can dominate the water–ice phase transition with network solidification typically present in small sample cryo-straws or cryo-vials. Progressive solidification is more often observed in larger volumes or environmental freezing. These different ice phase progressions could have a significant impact on cryopreservation in scale-up and larger volume cryo-banking protocols necessitating their study when considering cell therapy applications.

This study determines the impact of these different processes on alginate encapsulated liver spheroids (ELS) as a model system during cryopreservation, and develops a method to replicate these differences in an economical manner.

It was found in the current studies that progressive solidification resulted in fewer, but proportionally more viable cells 24 h post-thaw compared with network solidification. The differences between the groups diminished at later time points post-thaw as cells recovered the ability to undertake cell division, with no statistically significant differences seen by either 48 h or 72 h in recovery cultures.

Thus progressive solidification itself should not prove a significant hurdle in the search for successful cryopreservation in large volumes. However, some small but significant differences were noted in total viable cell recoveries and functional assessments between samples cooled with either progressive or network solidification, and these require further investigation.

## Introduction

A bioartificial liver (BAL) machine can temporarily replace the functions of the liver, allowing a damaged liver to regenerate while protecting the patient’s other organs from the life-threatening damage that ensues during liver failure. The technology for growing an immortalised hepatocyte cell line (HepG2), encapsulation in alginate beads and proliferating and conditioning of the cell spheroids within the beads has been demonstrated at the large scale. However, widespread uptake of the BAL technology can only realistically be achieved with cryopreservation as a component of the manufacturing strategy. On demand manufacture of the BAL is not feasible, neither on the basis of cost nor logistics. A single disposable cassette encompassing all processing steps (perfusion, cryopreservation, cell conditioning), would greatly simplify safety and regulatory requirements, provide robust delivery to end users, and facilitate safe delivery in the clinical environment. However, for clinical delivery of a BAL, cryopreservation of up to 2 l of alginate encapsulated cell spheroids (ELS) are required in a single treatment and these would be ideally contained within a cylindrical cell cassette resulting in a packed product depth of up to 70 mm in a cylindrical chamber of length 30 cm held horizontally. While there are reports of the cryopreservation in bags of large volumes (>100 ml) of adult stem cells [25], mammalian tissue culture cells [8], [9], [12] and ELS [15], the geometry of these samples have been those of a thin slab (2d sample) less than 20 mm in thickness. These experience lesser thermal gradients than in our system. The bulk cryopreservation of mammalian cells at a scale and format required for a BAL, or indeed other cell therapies, has not been extensively studied previously.

The physical determinants of the freezing process in either large or small volumes are fundamentally different. In low volume samples (e.g. in straws, or cryovials with volumes <2 ml) at the typical cooling rates used in cryopreservation only small temperature gradients tend to occur throughout the sample. The whole volume generally undercool in a uniform way, i.e. cooled below the equilibrium melting point (the highest temperature at which ice and water can co-exist in steady-state) before ice nucleation commences [18], [20], [21]. Following the initial ice nucleation, which can be induced by a nucleating agent [6], [7], growth of a continuous ice network throughout the whole sample occurs rapidly, resulting in a coexisting, continuous phase of freeze concentrated material in which the excluded solutes and cells are distributed [20]. As a result of the migration of water from the freeze concentrated matrix, this ice network grows as a coherent entity during subsequent cooling. The structure of the ice network and of the corresponding freeze concentrated matrix is determined by the nucleation temperature [3] and not the rate of cooling [24]. In materials science this solidification process is called cellular growth [26]; however in order to avoid confusion when considering cell cryopreservation in a biological context, in which cell growth refers to cell proliferation, we will refer to this mode of ice solidification as network (or dendritic) solidification (NS).

In bulk samples significant temperature gradients may exist between the cooling interface (often the outer surface of the sample) and the bulk volume unless infinitesimally slow cooling rates are applied. Localized undercooling can easily occur at the container wall while there remains a gradient in the bulk sample leading to temperatures remaining above the equilibrium melting point for a significant time [19]. Nucleation of ice will occur at the cold wall and ice will develop into the solution which was initially at a temperature above the equilibrium melting point. As cooling progresses across the sample and the ice nucleation temperature is achieved, an ice front perpendicular to the heat transfer vector front moves through the sample [23]. The structure of the ice front is determined by a number of factors including the nucleation temperature, the rate of heat extraction, and localized inhomogeneities in temperature across the ice front, further complicated by release of latent heat of the ice crystallization process [18]. Depending on the solute composition and the rate of growth of the ice front, solute rejection (including rejection of structures such as cells) can occur ahead of the advancing ice interface [5], [14]. In this configuration, only the small proportion of the sample in contact with the cold wall was initially undercooled to any significant degree. In metallurgy this mode of solidification is referred to as progressive or parallel solidification [26] and we shall refer to this as PS when considering ice formation.

In order to develop protocols rapidly and efficiently for the cryopreservation of large volumes it is necessary to develop and validate a scale down method to emulate the process of ice formation that occurs within a large volume in comparison to that within a standard cryovial. This approach allows multiple samples to be tested within the same run, and also the effects of thawing to be de-coupled from the freezing step which produces either PS or NS. We also designed a technique to reliably produce PS in small volumes, removing the compounding factor of sample volume on the ice solidification process. In this study, we examined the viability and cell function of ELS (where we have extensive previous experience of post-cryopreservation functional assessment) [15], [16], [17] following either PS or NS. In addition we determined, by CryoSEM, the structure of the ice crystal networks and the residual freeze concentrated matrix following water to ice phase transition by these two methods.

## Materials and methods

### Cell culture and encapsulation

The techniques for producing ELS have been described previously in detail [4]. HepG2 cells (human-derived hepatocyte cell-line) were grown in monolayer culture for 7 days and passaged at 80–90% confluence. Culture medium composed of alpha-MEM medium, supplemented with 50 U/ml penicillin, 50 μg/ml streptomycin (Invitrogen plc.), and 10% FCS (Hyclone Thermo Scientific). A suspension of 3.5 × 10^6^ cells/ml in culture medium mixed 1:1 with 2% aqueous alginate solution (FMC bio-polymers), was passed through a jetcutter system (GeniaLab), resulting in spherical droplets with a diameter of 500–550 μm, which were polymerised by ejection into a buffer with 0.204 M CaCl_2_. These (ELS) were grown in culture medium at a ratio of beads to medium of 1:32 in static culture (T175 flasks) in a 5% CO_2_ humidified incubator at 37 °C for 11 days, with medium changed every 2–3 days, where they proliferated to approximately 1 × 10^7^ cells/ml.

### Establishment of cooling profiles in a representative large scale volume containing an ELS thermal mimic

For typical PS in a true large volume experiment, a prototype of the cylindrical BAL cassette constructed out of polycarbonate and containing 2000 ml of a 10% glycerol in water (v/v) solution as an ELS thermal mimic was cooled on its side on a modified VIAFreeze controlled rate freezer (Asymptote, Cambridge, UK). Good thermal contact was achieved via a curved plate attached to the cassette (Fig. 2). To ensure good thermal contact between the cassette and the sample plate a film of low temperature silicone oil (Sigma, 85409) was applied to the sample plate. Thermocouples were placed throughout the chamber to measure thermal profiles in the ELS thermal mimic, using 10% glycerol which we have established previously has equivalent thermal properties to our alginate encapsulated biomass (data not shown).

### Modification of the controlled rate freezer to achieve NS or PS in small volumes during cryopreservation

**Fig. 1:**
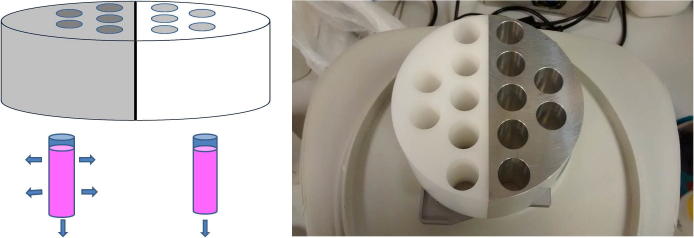
The two different heat transfer modules designed for the EF600-103 CRF. On the left an aluminum module allowed for maximum heat transfer between the vial and the insert, on the right the acetal insert only allowed for heat transfer between the bottom of the vial and the EF600-103 cooling plate. Both modules operated concurrently during cryopreservation, allowing the study of up to seven replicates in each condition, at identical cooling rates.

A controlled rate freezer (EF600-103, Asymptote, Cambridge, UK) was modified to achieve either NS or PS during cryopreservation by the addition of two modules designed to take polypropylene scintillation vials (Sigma, Z376825, 16 mm × 57 mm). One module was made of aluminum, the other of acetal (Fig. 1); these materials are good and poor conductors of heat respectively. These modules were fixed to the flat cooling plate of the EF600-103. Thermocouples (K type) we used to measure the temperature at the base, middle, and upper sample volume inside the vial, (0 mm, 20 mm, and 40 mm from base respectively) the thermocouples were connected to a Pico Logger (Pico-technology).

**Fig. 2:**
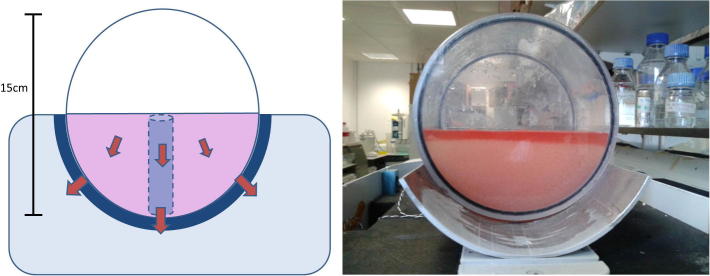
Schematic and image of the large volume cylindrical chamber. Excess media is drained out of the prototype chamber prior to cryopreservation and the resulting ELS thermal mimic (10% glycerol) fills the BAL chamber halfway, (the remaining upper volume containing air). The BAL chamber was then placed on a Stirling engine cooled metal plate (dark blue). The arrows indicate heat transfer during cryopreservation, while the dashed outline of a vial is overlain, indicating the approximate area modeled with the heat transfer modules in Fig. 1. (For interpretation of the references to color in this figure legend, the reader is referred to the web version of this article.)

### Cryopreservation protocol

For small volume PS or NS studies, 5 ml aliquots of ELS were harvested and mixed 1:1 with a freezing solution (24% Me_2_SO, 76% Viaspan v/v) precooled to 4 °C, and once equilibrated (15 min), 80% of the excess CPA supernatant was removed, giving a final volume of 6 ml of 12% Me_2_SO, 38% Viaspan, and 50% ELS in culture medium, by volume. Icestart beads (1% w/v) (Asymptote) – sterile insoluble granules – which induce ice nucleation close to the equilibrium melting temperature of the mixture, were added and these sank by gravity to the base of the vial. These vials and the CRF were cooled to 4 °C before 5 vials (containing 6 ml each) were placed in the aluminum module, while 5 were placed into the acetal module (see Fig. 1). The EF600-103 was programmed to cool at 1 °C/min from 4 °C to −80 °C. The samples were held in the EF600-103 at −80 °C for 1 h after the cooling cycle was complete, before being transferred to a −80 °C freezer for 7 days.

The samples were warmed rapidly during 330 s in a 37 °C water bath until all the ice had melted (yielding an approximate warming rate of 15 °C/min). The Me_2_SO was diluted out of solution during a 10 min stepwise process with prepared chilled culture medium, with residual ice start granules remaining at the bottom of the tube and easily avoided during decanting. The samples were re-cultured in a 5% CO_2_ humidified incubator at 37 °C.

### Cryo scanning electron microscopy (CryoSEM) of samples cooled by PS or NS

To observe physical changes in the structure of the samples, CryoSEM was carried out. Samples recovered from storage at −80 °C were warmed slightly (25 s in a 37 °C water bath) to loosen the ice matrix from the container wall, allowing the bulk frozen samples to be removed rapidly and transferred onto dry ice (−78 °C) without re-warming. These were wrapped in foil and stored on dry ice before being transferred to a −80 °C freezer.

Under liquid nitrogen, each sample was held in a metal bracket and split horizontally using a blade, giving a circular cross-section. This was transferred into a cryo scanning electron microscope (FEI XL30 FEGSEM with a Quorum pp2000 cryo-stage) and etched at −80 °C, before being coated in a thin layer (∼20 nm) of gold. The samples in the microscope and images were captured at 5 kV using an Everhardt Thornley secondary electron detector.

### Post-thaw functional tests after either PS or NS cryopreservation

#### Viability assay

A viability assay was carried out using PI/FDA staining. 20 μl PI (propidium iodine solution, 1 mg/ml, Sigma) and 10 μl FDA (fluorescein diacetate solution 1 mg/ml, Sigma) were added to ELS and incubated at room temperature for 90 s. The ELS were washed once in PBS (Invitrogen) and then florescence at 617 nm (excitation) and 520 nm (emission) measured, with 1 s and 150 ms exposure respectively. The total FDA intensity was compared to the total PI plus FDA intensity using Nikon imaging software, giving both a cell membrane integrity and metabolic viability read-out. This was carried out at 6, 24, 48, and 72 h post-thaw. The 6 h timepoint was chosen as this was the minimum time required to fully remove residual (pre-freeze) FDA-sensitive enzymes from non-viable cells.

#### Total cell counts

A known volume of ELS were removed from alginate post-cryopreservation in 16 mM EDTA (Applichem) solution before the ELS were dis-aggregated and a nucleic count carried out using the nucleocounter system. Since HepG2 cells are mononuclear this equates to cell number.

#### MTT assay

Further standardized samples of ELS were liberated from alginate and 0.75% w/v MTT solution (tetrazolium salt, invitrogen) added to the ELS. After 3 h incubation the MTT was removed and the crystal product dissolved using acidified isopropanol (10% acetic acid in propan-2-ol). Total absorbance was measured at 570 nm on an Anthos III microplate reader, and quantified using MANTA software.

#### Enzyme-linked-immuno-sorbent-assays (ELISA)

Albumin, alpha-anti-trypsin and alpha-fetoprotein protein production were quantified by ELISA in ELS conditioned media collected 1–3 days post-thaw, and normalized with cell counts. The normalization took two separate forms, one related to cell count post-thaw which showed the average function of the cells surviving cryopreservation. A second normalization determined average production based on number of cells cryopreserved – therefore even cells that were destroyed during cryopreservation were accounted for here.

### Statistics

To determine significance between samples cryopreserved either through NS or PS, a Welch’s *t*-test was performed. To determine significance between samples experiencing the same conditions during cryopreservation at different time points, a Student’s *t*-test was performed. Significance was determined as *p* < 0.05. Samples for cell functional analysis contained five replicates unless otherwise stated.

## Results

### Measurement of the thermal histories of the different cooling processes in both the large volume prototype chamber and the scale-down modules

**Fig. 3:**
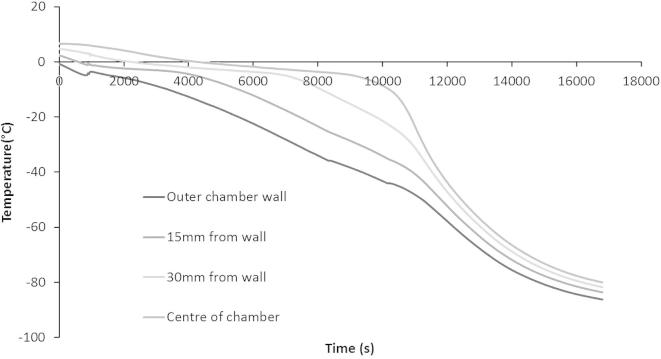
Measured temperature profiles inside the BAL chamber during cooling of a thermal mimic. Approximately 2000 ml of 10% aqueous glycerol solution (having the same thermal properties of ELS) was cooled in the large volume freezer, with pico-logger k-type thermocouples placed at progressively deeper intervals between the wall and the centre of the chamber, at 15 mm intervals. The freezer was programmed to cool from 4 °C to −80 °C at approximately −0.5 °C a min.

Measured temperatures within the large volume sample (Fig. 3) containing 10% glycerol in aqueous solution (v/v) show large temperature gradients between the wall of the cassette (in contact with the cooling plate) and the deeper (more central) layers of the sample. While the sample layer adjacent to the cylinder wall reduced in temperature approximately linearly, the central sample layers experienced delayed cooling, non-linearity of temperature change, and eventual solidification, with a temperature plateau existing in the core of the sample for some considerable time (in the region of 150–180 min) at the equilibrium melting temperature before solidification occurred.

**Fig. 4:**
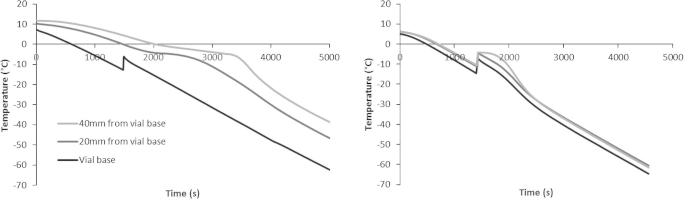
Temperature profiles in the heat transfer modules measured on the EF600-103 controlled rate freezer. K-type thermocouples were inserted at the base of the sample (which was placed on top of the cooling plate of the freezer) and at progressively deeper intervals. The temperature profiles were recorded in both the acetal module (left) and in the aluminum module (right). The EF600-103 was cooled from 4 °C to −80 °C at 1 °C/min, with samples containing 6 ml ELS in 12% Me_2_SO.

In the vials processed in the acetal or aluminum modules for the EF600-103, producing either PS or NS respectively, the monitored temperature profiles differed between the two processing conditions (Fig. 4). With vials in the acetal module, nucleation occurred at the bottom of the cryovial (again, next to the cooling plate of the cryo-cooler) where a small amount of undercooling is evident, while the remainder of the sample remained above the melting point of the solution. Ice growth occurred progressively (and in this case – vertically) within the remainder of this sample and no further significant undercooling was evident (see Fig. 4 – left) emulating the temperature profile, characteristic of progressive solidification seen in a large volume sample (Fig. 3). The whole of the sample volume within a vial in the aluminum module cooled uniformly below the equilibrium melting temperature of the solution before ice nucleation occurred and solidification then progressed instantaneously and in a relatively uniform manner throughout the cryovial, with no large temperature gradients being observed (Fig. 4 – right).

### Characterization of the ice morphologies in the different freezing processes in the scale-down modules

**Fig. 5:**
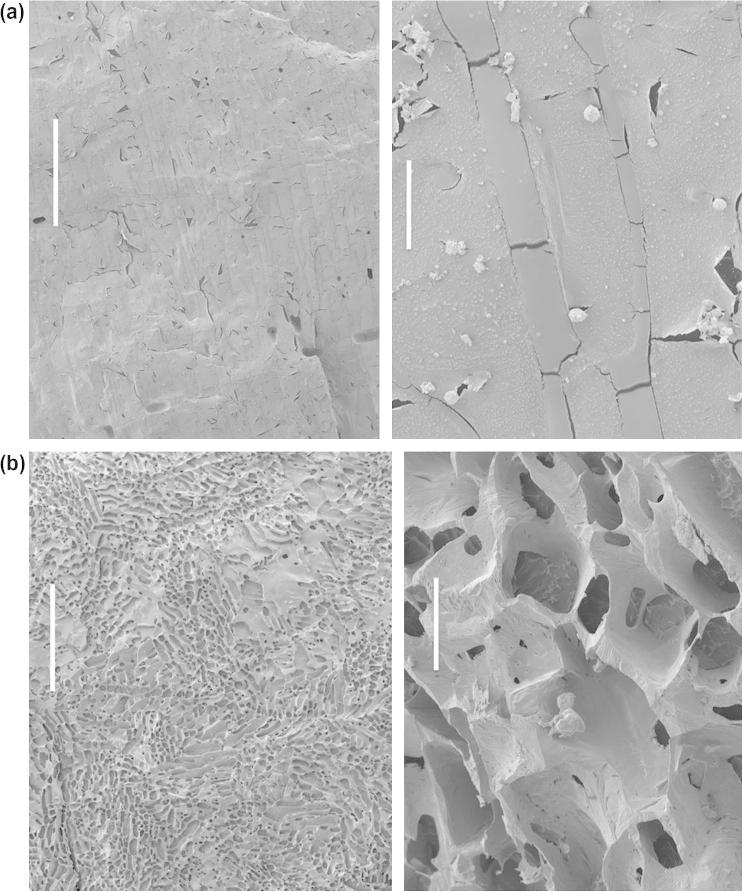
Cryo-scanning electron microscopy (Cryo-SEM) presenting differences in ice structure between progressive solidification (a) and a network solidification (b). The scale bar indicates 1 mm on the left hand images, while 50 μm on the right hand images in both cases. Progressive solidification exhibits a large and homogeneous ice crystal, indicative of slow growth during formation, while network solidification results in a profoundly separate, disordered dendritic ice structure.

The structure of the ice and the freeze concentrated matrix is very different in samples processed from vials within the two different modules where either NS or PS was developed (Fig. 5). A planer ice structure is present under conditions of PS in samples processed in the acetal module (Fig. 5A), with vertical ice crystals forming in the sample, entrapping ELS between ice crystals. Following NS (cooling in the aluminum module) a multiple dendritic (network) ice structure is apparent, with ice entrapping freeze concentrated matrix including ELS (Fig. 5B).

### Viability and viable cell number

**Fig. 6:**
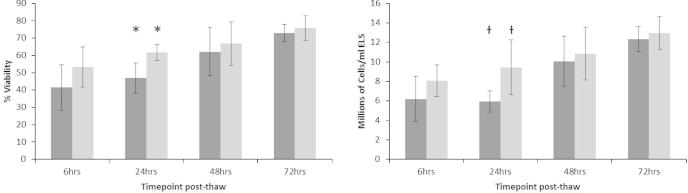
The cell membrane viability (left) and viable cell number (right) of samples experiencing either progressive solidification or network solidification during cryopreservation. Although network solidification (light gray) produces a better post-thaw outcome at 24 h (*p* < 0.05, *N* = 5 ± SD, marked by * and † for viability and viable cell number respectively), these differences disappeared by 48 h and 72 h in post-thaw cultures. Both PS and NS samples’ viability and viable cell number increased significantly from 6 h to 72 h (*p* < 0.05).

The cell viabilities, the viable cell numbers were quantified following either NS or PS at 6, 24, 48, and 72 h post-thaw (Fig. 6). The samples processed in the aluminum module (NS), displayed a trend towards higher average viability at all time points compared with samples processed in the acetal module; significance was noted for 24 h (*p* < 0.05, *n* = 5). The viabilities in both sample sets then further recovered and increased significantly (*p* < 0.05) with length of time in culture post-thaw out from 6 h to 72 h, from 53.2 ± 11.5% to 75.8 ± 7.1% and from 41.4 ± 13.1% to 72.8 ± 5.1% for the samples experiencing either NS or PS respectively. A similar pattern was true for total viable cell numbers (Fig. 6 – right) increasing significantly from 8.1 ± 1.6 to 13.0 ± 1.7 million cells/ml following NS. For samples from PS, they recovered significantly from a nadir at 24 h – 5.9 ± 1.1 million cells/ml to a maximum of 12.3 ± 1.3 million cells/ml at 72 h post-thaw; thus PS was significantly worse at 24 h (*p* < 0.05, *n* = 5) but not different by 72 h.

### Metabolic activity

**Fig. 7:**
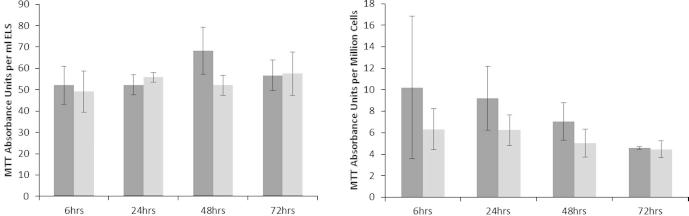
Metabolic activity in ELS: MTT absorbance units per ml ELS (left), and per million cells (right), between samples experiencing either progressive solidification or network solidification during cryopreservation. No statistically significant differences between the samples were observed at any time-point. No significant differences in MTT absorbance were observed intra-sample set between 6 and 72 h. *N* = 5 ± SD.

Metabolic activity of the samples post-thaw was analyzed using MTT. This was related to either the production per unit ELS (Fig. 7 – left), or to a viable cell number (Fig. 7 – right) and so function per cell – at 6, 24, 48, and 72 h post-thaw could be calculated. This is an important comparator to identify differences between cell populations from different culture batches. MTT metabolism per unit ELS (Fig. 7 – left), showed no significant difference between either NS or PS samples. When the MTT metabolism was expressed per million viable cells (Fig. 7 – right), the mean production per cell number appeared higher in PS compared with NS at all time points, although not reaching significance (*p* > 0.05, *n* = 5, in each case).

### Protein synthesis

Sandwich ELISAs determined protein production per million cells per 24 h in samples collected 1–3 days post thaw. Of the three quantified proteins, Alpha-fetoprotein (AFP) did not exhibit a significant difference at any time point.

In contrast, albumin production in the PS samples was significantly higher (*p* < 0.05, *n* = 5) 24 h post-thaw being measured at 46.7 ± 11.5 μg per million viable cells per 24 h, compared to 30.9 ± 4.4 μg per million viable cells per 24 h following NS.

Alpha-antitrypsin was also significantly improved (*p* < 0.05, *n* = 5) 24 h post thaw, at 18.8 ± 4.8 μg per million viable cells per 24 h, compared to 12.2 ± 2.0 μg per million viable cells per 24 h following NS.

**Fig. 8:**
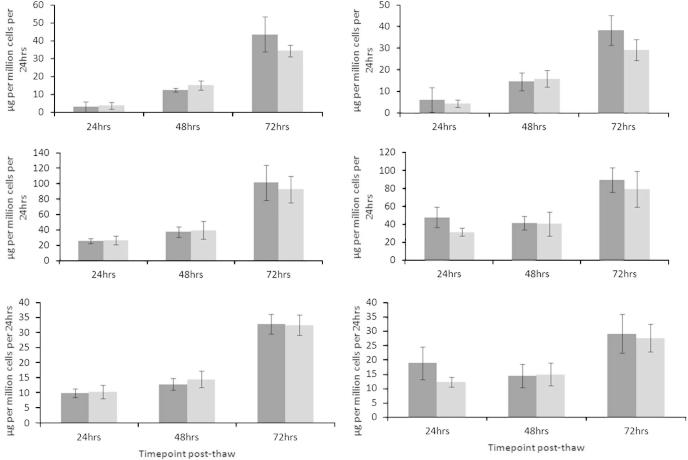
The production of liver specific proteins by ELS, day 1 to day 3 post-thaw between samples experiencing either progressive solidification or network solidification during cryopreservation – alpha-fetoprotein (top), albumin (middle), and alpha-antitrypsin (bottom). Progressive solidification samples are shown dark in gray with network solidification samples lighter gray. Production was analyzed per million viable cells post-thaw per 24 h (right), and normalized per million viable cells pre-thaw that takes account of cells destroyed during the cryopreservation (left). Measurements on the left therefore present the overall outcome per sample cryopreserved – there are no statistically significant differences. Measurements on the right display production per million cells, but taking into account that there is now a different cell number between the two sets. Allowing for this different cell number, the progressive solidification samples have significantly improved production of albumin and alpha-antitrypsin in the first 24 h post thaw (at *p* < 0.05). There are no significant differences at other time points or with alpha-fetoprotein *N* = 5 ± SD. Intra either NS or PS sample sets, the measured protein production increased significantly from day 1 to day 3 for all measurements (*p* < 0.05).

All protein production capabilities in either NS or PS samples improved significantly from 24 h to 72 h post-thaw, mirroring the recoveries in viable cell numbers during progressive post-thaw culture (see Fig. 8).

## Discussion

Ice solidification occurs in small and large volumes by two distinct processes. At small volumes network solidification (NS) manifests while at large volumes progressive solidification (PS) is the predominant process. These differences in bio-physical events presented as different ice crystal formats in this study. Similar differences in ice matrix ultrastructure have been presented for sperm processed either in straws or bags [22].

With ELS, the observed recovery following these two processes was very similar although the structure of ice and the freeze concentrated residual compartments within the two types of samples are very different. Post-thaw, samples experiencing NS had a higher post-thaw viability and viable cell numbers, significant after 24 h of recovery. When examining the functional outcomes, samples cryopreserved experiencing PS have an improved outcome per unit of viable cells, although overall differences were small. Our results suggest that NS allows more cells to survive cryopreservation, but those surviving cells have greater average damage than those experiencing PS. PS by contract showed a trend to fewer, healthier cells post thaw, especially at the 24 h time point following thawing.

During large scale cryopreservation the potential long exposure to cryoprotectants in the liquid state prior to phase transition, experienced for the central portion of the sample under condition of PS, may be a potential extra stress over and above those which result from cryopreservation in NS conditions. We had considered the possibility that PS would therefore result in greatly reduced post-thaw recoveries. This was in fact not the case, which is encouraging when planning further work on scaled-up cryopreservation in volumes >1 l. It could be hypothesised that under conditions of PS, the extra cryoprotectant stress experienced by part of the sample could act to remove an unhealthy, or poorly performing sub population of cells present before cryopreservation. NS, by reducing the time to which the ELS from the whole sample was exposed to the osmotic and chemical toxicities, where the central mixture was in the liquid state just at the point of nucleation, may avoid injuring this already partially stressed population leading to significantly higher viable cell numbers (although metabolically less productive) by 24 h post-thaw. It is also possible that the temperature discontinuity present when an undercooled sample nucleates damages cells in subtle ways, so they survive cryopreservation though are no longer function effectively. Further studies will need to investigate these mechanisms.

It is important to differentiate the processes described above (NS and PS) from another way to control ice crystal progression – this being the so-called directional solidification (DS) where the sample is moved across a constantly low temperature gradient, sufficiently cold to induce ice nucleation in the portion of the sample in contact with the cold plate. DS allows the morphology of the ice interface to be varied under conditions where the local chemical conditions of the residual solution can be kept constant, which is different to what happens in PS where progressive exclusion of both solutes and cells occurs ahead of the ice front. The technique allowed investigation of whether different ice crystal morphologies (for example, with increasingly complex ice dendrite formation) impacted on cell survival, but this was not generally found to be the case [10]. Differential entrapment or exclusion of cells within the advancing ice front was also noted with DS [11], but the behavior of larger cell complexes (such as the ELS) has not been investigated as far as we are aware. PS would perhaps be expected to deliver ice fronts moving between and through the alginate capsules containing the ELS, which were used in relatively high packing density in the current study, but further work will be needed to investigate this aspect. DS also allows better homogeneity of the cooling profile throughout the entire sample [2], whereas, as seen here, PS results in differential thermal profiles towards the sample centre as the excluded solutes, generating areas of local undercooling, result in variable release of latent heat of ice crystal formation. This heat has to be dissipated from the sample core before linear cooling can proceed. This results in a controllable progression of solidification through the specimen dependant on the rate at which the temperature gradient is passed through the sample [1], [10], [11]. DS allows the morphology of the ice interface to be varied under conditions where the local chemical conditions of the residual solution can be kept constant, which is different to what happens in PS where progressive exclusion of both solutes and, in some situations, cells occurs ahead of the ice front [11]. DS also allows better homogeneity of the cooling profile throughout the entire sample, whereas, as seen here, PS results in differential thermal profiles towards the sample centre as the excluded solutes, generating areas of local undercooling, result in variable release of latent heat of ice crystal formation which have to be dissipated from the sample core before controlled cooling can proceed. However, for large cell masses contained within an irregular geometry as investigated here, engineering a DS approach to cryo-cooling would prove to be challenging. In the current work, solidification proceeded only through static surface cooling conditions, with ice growth primarily determined by the thermal properties and 3-dimensional structure of the sample.

Another factor worthy of comment is that the experimental systems used here had little excess cryoprotectant additive and there would be little settling effect of ELS on the ice crystal progression – all the samples were in effect ‘settled’ by removing the extra CPA volume. The process of ice propagation in this system may differ compared with conventional cell and protein suspensions, where sedimentation of cells may occur before initiation of freezing and, secondly, cells and proteins may be pushed ahead of ice fronts during progressive solidification.

While success has been reported with large volumes in flat bag cryopreservation, these have generally been deliberately compressed into a thin wafer or ‘slab’ format with little internal temperature gradients and so often experience NS. It is possible to observe PS in bags however, if the bag temperature is not thermally equilibrated prior to the onset of solidification [15], [25].

Such flat-bag approaches would be very difficult to adapt for BAL cryopreservation due to the geometries involved, where the end-product would ideally reside in a cylindrical fluidised bed format.

The varying temperature profiles throughout the sample when cooling a large cylinder have been recognized for some time [19]. Previous studies have shown that the level of freeze-concentration of solutes is dependent on the cooling rate and this has been studied in detail in cylindrical vessels [13]. In cylindrical configurations, the solutes increased in concentration radially from the edge of the cylinder to the centre, and this was accompanied by aggregation of some proteins within the core layers. Due to the alginate sphere composition of the test BAL, cell aggregation will not occur here as the cells are already immobilised. This increase in solutes centrally (as would be seen in a cylindrical BAL cassette) is a likely cause of lower cell number post thaw when progressive solidification is present in our current scale-down module.

Using inserts in the EF600-103 to emulate large volume cooling profiles within small samples gave similar thermal histories as were seen in a large volume. This allowed for the study of these thermal profiles as well as longer and variable cryoprotectant exposure and cryo-concentration of solutes in the system, in addition to accurately mimicking the variations in ice structure between the two set-ups. Combining these three effects in a smaller volume format accurately provides more accessible and more economical methods of study of these sample configurations, without the additional variable of differing volume or thawing rate. This equipment modification may have application in studying other large volume freezing problems, such as those encountered with proteins.

Significantly this study informs us that PS may be applied to the BAL without major detrimental effects on the bulk ELS product, although there was a low level of early functional attrition seen after PS which requires further study.

## Conclusion

Previously our group reported good outcome when ELS (cryopreserved in typical small volume format in cryo-vials) experienced network solidification during cryopreservation [16], [17]. Good outcomes can now be achieved in a more realistic large scale geometry that necessarily produces progressive solidification, and this can be modeled in an economical way using an adapted head plate for the EF600-103 freezer.

It has been demonstrated that both PS and NS exhibit very different biophysical conditions during ice crystal growth; this is reflected in the ultrastructural observations of the differing ice-matrices during solidification. However these different outcomes of cryo-solidification in reality made only small, mostly non-significant differences to viable cell recovery or function. ELS cryopreserved under both conditions each showed very good propensity to return to normal cell replication as post-thaw culture extended beyond the first 24 h. As progressive solidification is almost unavoidable in samples any larger than a few mls, an understanding of the differences between these two conditions may well be necessary for successful larger volume cryopreservation across a wide range of cell therapies.
